# Compassion focused therapy for women in the perinatal period: a summary of the current literature

**DOI:** 10.3389/fpsyt.2023.1288797

**Published:** 2023-12-19

**Authors:** Leah Alice Millard, Anja Wittkowski

**Affiliations:** ^1^Division of Psychology and Mental Health, School of Health Sciences, The University of Manchester, Manchester, United Kingdom; ^2^Greater Manchester Mental Health NHS Foundation Trust, Manchester, United Kingdom; ^3^Manchester Academic Health Science Centre, Manchester, United Kingdom

**Keywords:** compassionate, maternal mental health, self-compassion, psychological therapy, parent-infant relationship

## Abstract

**Introduction:**

Compassion focused therapy (CFT) is emerging as an effective psychological intervention to treat those experiencing mental health difficulties. CFT was adapted for women who are mothers during the perinatal period (from conception to 2 year postpartum). Although CFT is being delivered in NHS perinatal mental health services in the United Kingdom (UK), its current evidence-base for the treatment of women’s mental health problems is unclear. As part of this Mini Review, we aimed to identify the current findings relating to CFT for women in the perinatal period (with or without a mental health condition) in order to identify any associated future research and clinical implications.

**Method:**

A systematic search of two databases was undertaken. Included studies were required to meet the following criteria: (1) offered an intervention using CFT or perinatal CFT (P-CFT), (2) participants were women in the perinatal period, and (3) studies used a pre- and post-intervention study design. No language restrictions were used. A narrative synthesis was then conducted.

**Results:**

Five studies, dating from 2018 to 2023, met the inclusion criteria. A total of 1,258 participants were included across those studies. Significant improvements in compassion-based outcomes (i.e., self-compassion, self-criticism/self-reassurance) were observed. However, these findings were primarily derived from non-clinical samples (*n* = 4) and could only be seen as preliminary.

**Conclusion:**

Although these results are encouraging for mothers presenting with sub-clinical mental health symptoms, further research is clearly warranted to determine whether CFT/P-CFT may benefit mothers, including those presenting with more significant perinatal mental health difficulties.

## 1 Introduction

Around 10–20% of women experience perinatal mental health (PMH) difficulties, which occur either during pregnancy or within the first year postpartum (1). PMH difficulties can vary widely in severity (i.e., mild, moderate, or severe) and can span a wide diagnostic spectrum (2). For instance, anxiety and depression are prevalent issues during pregnancy, affecting around 15–20% of expectant mothers (2, 3). A lower proportion of women may be at risk of onset or recurrence of a severe mental illness (SMI) during the early postpartum period, with one to two women per 1,000 births requiring psychiatric admission shortly after giving birth (2, 4).

Depending on the severity of her PMH condition, a woman may require different treatment approaches, including pharmacological, psychosocial and/or psychological ones. In the UK, the NICE (5) guidelines recommend pharmacological approaches for moderate to severe PMH difficulties, but during pregnancy NICE (5) recommend psychological approaches because of potential adverse effects associated with pharmacological interventions. At present, the body of evidence for psychological interventions for treating PMH difficulties is confined to cognitive behavioral therapy (CBT) or interpersonal psychotherapy (IPT) (6). Therefore, CBT and IPT are the current recommendations in the UK perinatal competency framework (7). This recommendation is partly due to the limited number of research into the effectiveness and acceptability of other psychological approaches. Other interventions are offered to address a particular diagnosis (e.g., psychosis-related disorders, bipolar-related disorders) (5), but these interventions have not been specifically adapted for or tested in women with PMH conditions.

Although CBT can clearly improve outcomes in postnatal depression (8), there is less evidence for the intervention targeting more than the women’s PMH difficulties, including her relationship with her baby (9, 10). Psychological interventions offered specifically to mothers should address the mother-infant bond and relationship (5). A poor mother-infant relationship can have adverse outcomes for a child’s long-term emotional, social, and cognitive development (11).

Other therapies are currently being offered in perinatal mental health community teams in the UK that appear to be suitable in addressing both the maternal mental health difficulties, as well as improving the mother-infant relationship (12). One such therapy is Gilbert’s (13, 14) compassion focused therapy (CFT), which has been adapted by Cree (15, 16) for PMH difficulties. However, this intervention has not yet been extensively studied in those experiencing PMH difficulties (6).

### 1.1 Perinatal compassion-focused therapy

Compassion focused therapy (CFT) is a transdiagnostic intervention that differs from other compassion-based and mindfulness therapies [e.g., compassion cultivation training (17), mindful self-compassion program (18)], because it is grounded in a broad spectrum of psychological approaches ranging from neuroscience to Buddhist psychology (7). CFT aims to achieve a balance between the three emotional regulatory systems known as the threat system (fight-or-flight), drive system (reward and excitement), and the soothing-oxytocin system (calmness and self-compassion). CFT was developed for individuals with high levels of self-criticism, who are theorized to have an overriding threat system, and a suppressed soothing system (13, 14). Service-users with mental health conditions in clinical settings are often characterized by high levels of shame and self-criticism, including those in the perinatal period (15). For example, in their longitudinal study of 32 pregnant women attending an antenatal clinic, Brassel et al. (19) reported significant increases in levels of self-criticism from pregnancy to the postpartum period. Using the self-report questionnaire maternal postnatal attachment scale (MPAS), (20), lower levels of self-criticism at 30 weeks’ gestation were reported to be associated with an absence of hostility toward the infant at 18-month-postpartum (19). Therefore, it is likely that higher levels of self-criticism during pregnancy may have negative consequences on the mother-infant bond. For this reason, it may be important to address the mother-infant relationship in psychological interventions that are offered to mothers.

Compassion focused therapy (CFT) has previously been reviewed in the literature using different parameters including non-clinical groups (21, 22), comparisons with other compassion-based models (22), mixed study designs (21, 23), and clinical groups (23). The most recent systematic review focussed on Gilbert’s (13, 14) CFT when offered to clinical groups of participants only (e.g., major depressive disorder, eating disorders, etc.). In their review and meta-analysis of 15 eligible studies, Millard et al. (24) found that CFT led to significant improvements in compassion-based outcomes, such as self-compassion and self-reassurance, and a reduction in levels of self-criticism and clinical symptomology. Although no eligible studies including women in the perinatal period were identified in the search, approximately 75% of participants across all of the 15 included studies identified as women.

Clinical psychologist Cree (15, 16) adapted CFT specifically for mothers within the perinatal period to address their feelings of shame and self-criticism that could have arisen in their role as mothers and to reduce any potential bonding difficulties.^1^ Cree (15, 16) theorizes that a suppressed soothing-oxytocin system may hinder the mother-infant relationship, with oxytocin being an important hormone for promoting mother-infant bonding. Therefore, Cree (15) explains that the first part of P-CFT seeks to develop a mother’s self-compassion by activating the soothing-oxytocin system through techniques known as compassionate skills (i.e., attention, imagery, reasoning, behavior, sensory, feeling), so individuals can develop compassionate attributes (i.e., sensitivity, sympathy, distress tolerance, care for wellbeing, empathy, non-judgement). These attributes are thought to enhance a mother’s ability to bond with her infant. The term bonding can be defined as the emotional connection from the parent to the infant that is typically marked by the parent’s feelings and emotions toward the infant (25).

Perinatal compassion focused therapy (P-CFT) introduces mothers to seven overlapping attributes to strengthen the mother-infant relationship and/or address any bonding difficulties: (1) motivation to care for infant, (2) warmth toward infant, (3) non-judgmental acceptance of infant, (4) emotional regulation, (5) attunement, (6) maternal sensitivity, and (7) maternal mind-mindedness (15). Cree (15) explained how these maternal attributes could alter the mother-infant relationship. The motivation to care for infant wellbeing refers to a mother changing the perception of her relationship with her infant. This change involves switching from a threat-associated motivation (i.e., the mother protecting themselves or her infant from the other) to a compassion-based motivation by encouraging child development through caring for and soothing them. To do so, the mother develops warmth toward the infant, non-judgemental acceptance and facilitates mutual emotional regulation through increasing proximity (e.g., implementing compassion-focused techniques) (15).

As well as conveying physical closeness and protection, mothers seek to be attuned to her infant’s needs through positive responses (15). Attunement often involves emotionally regulating her infant to reduce initial signs of discomfort through techniques such as changing facial expression or tone of voice. To be attuned to an infant’s needs, maternal sensitivity is required. A lack of maternal sensitivity often presents itself through either a mother being unresponsive to her infant’s needs or when a mother’s attempts to influence her infant’s behavior based on her own mind. These two attributes enable the mother to develop maternal mind-mindedness, wherein a mother can view her infant as having their own mind, feelings thoughts and beliefs that differ from her own (15).

Perinatal compassion focused therapy (P-CFT) has gained prevalence with healthcare professionals and clinicians in perinatal mental health (PMH) settings in the UK (6), with group delivery of this intervention being common, especially in England. After conducting our review and meta-analysis (24), the dearth of studies exploring the effectiveness of CFT or P-CFT in women and mothers in the perinatal period became evident. Consequently, we set out to explore whether the literature on CFT or P-CFT was emerging for this particular epidemiology. Secondly, as this intervention was specifically designed to address both PMH difficulties and the mother-infant bond, it is important to determine the existence of any research into these specific outcomes.

Therefore, this Mini Review focused on (a) identifying published research studies that offered either Gilbert’s (13, 14) model of CFT or Cree’s (15, 16) P-CFT adaptation to women in the perinatal period, irrespective if drawn from clinical or non-clinical samples, and (b) on examining the benefits of this type of intervention in terms of reductions in mental health symptomology and/or improvements in the mother-infant bond.

## 2 Methodology

### 2.1 Search strategy

A literature search was performed across two databases: Web of Science and PubMed. Previous reviews on Gilbert’s (13, 14) model of CFT informed the search terms (23, 24). The terms and Boolean operators were as follows: “*compassion*” OR “*compassionate*” OR “*compassionate mind*” OR “*compassion-focused*” AND “*treatment*” OR “*therapy*” OR “therap*” OR “*training*” OR “*intervention*” AND “*perinatal*” OR “*postpartum*” OR “*postnatal*” OR “*pregnant*” OR “*pregnancy*” OR “*maternal*” OR “*mother*” OR “*antenatal*”. A search of “*perinatal compassion focused therapy*” on Google Scholar was also conducted.

### 2.2 Inclusion and exclusion criteria

The criteria for inclusion in this Mini Review were that a study must have: (1) delivered CFT that derived from either the work of Gilbert (13, 14) or Cree (15, 16) AND (2) participants that were women in the perinatal period AND (3) utilized a pre- and post-intervention data collection. No language restrictions were implemented.

## 3 Results

### 3.1 Study characteristics

Overall, five studies were identified (see Table 1). Four were retrieved through the systematic search (26–29), and one being identified through personal communication with the author (30). The studies were conducted across four countries, including the UK (*n* = 2), Australia and New Zealand (*n* = 2) and the USA (*n* = 1) between the years 2018 and 2023. Despite not limiting the search in terms of language, all studies were written in the English language.

**TABLE 1:** Overview of the five studies included in this review presented in chronological order.

	References Location	Design	Sample		Interven-tion (Reference)	Comparator/ control group	Retention rate	Delivery format and duration	Outcome measures		Main outcome(s)
			**Sample type**	**Included sample**					**Compassion-based**	**Other**	
1	(26) USA	Pilot RCT	Non-clinical sample of women who are pregnant, became pregnant within the last 12-months, and intend to become pregnant in the future	*n* = 137 (CMT *n* = 69; CBT *n* = 68)	Compassionate mind training (CMT) (13, 15, 16)	A “micro-intervention” of cognitive behavioral therapy (CBT) from Palo Alto University’s Institute of International Internet Interventions (i4health)	Overall: 65.0% (*n* = 89) CMT: 62.3% (*n* = 43) CBT: 67.6% (*n* = 46)	Self-guided online format. Details: 45-min didactic course that explained the course materials. Participants would then have access to the follow-up materials which were either CMT audio meditations or CBT exercises for two-weeks.	FSCRS (31) SCS-SF (32)	PHQ-4; (46) GAD-2 (47)	Similar improvements in compassion-based outcomes and affect across CMT and CBT. Greater significant reductions in depression (CMT: *M* = 1.07, SD = 0.27; CBT: *M* = 1.42, SD = 0.29, *p* = 0.03) and anxiety (CMT: *M* = 1.00, SD = 0.26; CBT: *M* = 1.49, SD = 0.26, *p* = 0.04) in CMT than CBT over time. Women who screened positive for depression at baseline significantly more likely to score below the depression cut-off point at follow-up in CMT group than CBT group (*p* = 0.04). No significant group difference for positive baseline screening of anxiety. No effect sizes reported.
2	(27) Australia and New Zealand	Feasibility and acceptability study (pre-intervention, 1-month post-intervention)	Non-clinical sample of mothers (< 24 months postpartum)	440	CFT-based resources (Authors of study)	Not applicable	59.5% (*n* = 262)	Self-guided online format. Details: two videos and a tip sheet. Unlimited access to resources for 1 month. One video and the tip sheet covered self-compassion in motherhood. The second video was a guided self-compassion visualization exercise.	SCS-SF [Raes et al. (32)]	AAQ-II (43) IES-R (44) MBFES (45) Other as shamer scale (OAS) (48)	49.8% accessed some or all of the resources. Small significant increase in self-compassion from baseline (*M* = 2.89, SD = 0.81) to post-intervention (*M* = 3.00, SD = 0.80; *p* = 0.002). Reported effect size was small (*d* = 0.11). No changes in psychological flexibility, shame, or satisfaction with breastfeeding
3	(28) Australia	RCT	Non-clinical sample of mothers (< 24 months postpartum)	*n* = 470 (intervention *n* = 231; waitlist control = 239)	Brief online self-compassion intervention (27)	Waitlist	Overall: 52.8% (*n* = 248) Intervention: 40.7% (*n* = 94) Waitlist control: 64.4% (*n* = 154)	Self-guided online format. Details: two videos and a tip sheet. Unlimited access to resources for 2 months. See Mitchell et al. (27).	Fears of compassion scales (FCS) (33) Compassionate engagement and actions scale (CEAS) (34)	DASS-21 (42) Acceptance and action questionnaire (AAQ-II) (43) Impact of event scale-revised (IES-R) (44) Maternal breastfeeding evaluation scale (MBFES) (45)	Significant intervention effects for compassion-based outcomes and affect compared to waitlist control at post-intervention: CEAS subscales for self-compassion engagement: CFT: *M* = 27.14 (SD = 6.56); Waitlist: *M* = 24.01, (SD = 7.44). Small effect size reported (η2 = 0.03) DASS 21 depression: CFT: *M* = 2.71, SD = 2.64; Waitlist: *M* = 3.98, SD = 4.22. Small effect size reported (η2 = 0.02). Posttraumatic stress symptoms: CFT: *M* = 5.68, SD = 6.81; Waitlist: *M* = 8.56, SD = 12.45. Small effect size reported (η2 = 0.02)
4	(29) UK	RCT	Non-clinical sample of mothers (< 12 months postpartum)	*n* = 206 (intervention *n* = 105; waitlist-control *n* = 101)	Kindness for Mums Online (KFMO) (13, 16, 39–41)	Waitlist	Overall: 65.5% (*n* = 135) KFMO: 51.4% (*n* = 54) Waitlist control: 80.2% (*n* = 81)	Self-guided online format. Details: a five-session interactive web-based program. Required duration for each weekly session was 10–15 min per week, and a few minutes of a daily exercise.	Self-compassion scale (SCS-SF) (32) FSCRS (31)	WEMWBS (35) Depression, anxiety and stress scales-Short form (DASS-21) (42)	Significant greater increases in KFMO group compared to waitlist control in self-compassion from baseline KFMO: *M* = 2.55, SD = 0.69; Waitlist: (*M* = 2.66, SD = 0.58) to post-intervention (KFMO: *M* = 2.94, SD = 0.63; Waitlist = *M* = 2.74, SD = 0.67) and from baseline to 6-week follow-up (KMFO: *M* = 3.05, SD = 0.67; Waitlist: *M* = 2.83, SD = 0.73). Small effect sizes reported.
5	(30) UK	Pre- and post-intervention	Clinical sample of mothers within a NHS IAPT service	*n* = 5	P-CFT (16)	Not applicable	75% (*n* = 4)	Eight-session group led by therapy facilitators (in-person)	Forms of self-criticism/self-attacking scale (FSCRS) (31)	Patient health questionnaire-9 (PHQ-9) (37) Generalized anxiety disorder-7 (GAD-7) (38)	Decreases in self-criticism and improvements in self-reassurance. Reductions in anxiety and depression scores. No inferential statistics reported.

AAQ-II, acceptance and action questionnaire (43); CEAS, compassionate engagement and actions scale (34); DASS-21, depression, anxiety and stress scales-short form (42); FCS, fears of compassion scales (33); FSCRS, forms of self-criticism/self-attacking scale (31); GAD-2, generalized anxiety disorder-2 (47); GAD-7, generalized anxiety disorder-7 (38); IES-R, impact of event scale-revised (44); MBFES, maternal breastfeeding evaluation scale (45); OAS, Other as shamer scale (48); SCS-SF, self-compassion scale-short form (32); PHQ-4, patient health questionnaire-4 (46); PHQ-9, patient health questionnaire-9 (37); WEMWBS, the Warwick-Edinburgh Mental Wellbeing Scale (35).

A total of 1,258 participants were included across those five studies. Sample sizes ranged from five (30) to 470 (28). The overall retention rate from baseline to post-intervention was 58.7% (*n* = 738), ranging from 52.8% (28) to 75.0% (30). The retention rate for the CFT intervention was 53.8% (*n* = 457) at post-intervention. Griffiths and Virgin (30) conducted the only study with a clinical sample of mothers experiencing PMH difficulties, whereas the remaining four studies used samples of mothers from the general population (26–29).

Three studies used a RCT as their chosen design (26, 28, 29). Mitchell et al. (27) and Griffiths and Virgin (30) adopted a pre- and post-intervention design with no comparator group. Four of the five identified studies delivered the therapy as a self-guided online intervention (26–29), whereas Griffiths and Virgin (30) was the only study that delivered treatment with a group format. All study authors asked participants to complete administered self-report questionnaires for their outcome measures. Four compassion-based questionnaires were used, namely the forms of self-criticism/self-attacking and self-reassurance scale (FSCRS) [(31), used in (26, 29, 30)], Raes et al.’s (32) self-compassion scale-short form (SCS-SF) [used in (26, 27, 29)], Gilbert et al.’s (33) fears of compassion scale (FCS) [used in (28)], and the Compassionate Engagement and Action Scale (CEAS) [(34), used in (28)]. Although other measures were used (see Table 1), none of the five study authors measured changes in the mother-infant relationship.

### 3.2 The current evidence of P-CFT

The five studies that have been identified in our search warrant further investigation. Kelman et al. (26) conducted a pilot randomized trial which compared two brief online interventions of CBT with compassionate mind training (CMT) in 123 women who were either currently pregnant, had been pregnant recently, or intending to become pregnant. Results revealed that both interventions were similar in improving compassion-based outcomes (see Table 1). However, the CFT-based intervention (*n* = 61) showed greater significant reductions in women’s symptoms of anxiety (*p* = 0.04) and depression (*p* = 0.03) compared to CBT (*n* = 62). No effect sizes were reported. The study recruited from the general population; however, subgroup analyses were conducted of the women who screened for anxiety (*n* = 31) and/or depression (*n* = 27) at baseline. The women randomly allocated to the CMT group who screened positive for depression were significantly more likely to score below the depression cut-off score at two-week follow-up than the CBT group (*p* = 0.04). No group differences were found in women who initially screened positive for anxiety at baseline.

Adapting Gilbert’s (13, 14) model of CFT, Mitchell et al. (27) delivered a self-guided online intervention to a non-clinical sample of 262 mothers who were within a 24-month postpartum timeframe. The brief self-compassion intervention consisted of two online videos, which involved (1) psychoeducation on self-compassion in the context of motherhood and (2) a CFT visualization exercise, and a self-help tip sheet promoting self-compassion. From baseline to a one-month post-intervention follow-up, the authors noted a small significant increase in self-compassion with a small effect size (*p* = 0.002, *d* = 0.11; see Table 1). Subsequently, measuring the effectiveness of Mitchell et al.’s (27) online intervention in a randomized controlled trial (RCT) in another non-clinical sample of 248 mothers, Lennard et al. (28) noted significant improvements in compassionate measures and mood symptoms in the 94 mothers who were offered the intervention materials, compared to waitlist control (see Table 1). All reported effect sizes were small (ranging from η2 = 0.002–0.030).

These findings for this brief CFT intervention are encouraging for mothers presenting with self-reported and sub-clinical mental health symptoms. However, it remains to be seen if this brief intervention is effective in mothers presenting with more significant PMH difficulties.

Using another RCT design, Gammer et al. (29) compared the effectiveness of a low-intensity online programme known as Kindness for Mums Online (KMFO), which was compared against a waitlist control. The KMFO intervention combined several compassion-based and mindfulness models, including those by Gilbert (13) and Cree (16). Over six sessions, this intervention aimed to improve levels of compassion and mental health outcomes in a non-clinical sample of 206 UK-based mothers in the perinatal period. Although the intervention significantly improved levels of self-compassion (*p* = 0.017) in mothers in the intervention group (*n* = 105), compared to waitlist control (*n* = 101), high attrition rates were observed in the intervention group (e.g., 48.6% did not complete post-intervention measures) and the effect size was small. Furthermore, no significant changes were found on secondary measures including self-criticism, self-reassurance and mood. Interestingly, findings indicated that KMFO was more beneficial to those who had lower levels of wellbeing at baseline, as measured by the Warwick-Edinburgh Mental Wellbeing Scale (35). The findings by Gammer et al. (29) are encouraging given that their intervention was partly based on P-CFT and that women in the intervention group reported improvements in their levels of self-compassion, even if no other measures were significant (see Table 1).

Griffiths and Virgin (30) conducted the only study that used Cree’s model of P-CFT only and/or included a clinical sample (i.e., receiving treatment for mental health difficulties) of mothers in the perinatal period. However, this study used a very small sample size (*n* = 5) that implemented a pre- and post-intervention design with no control group. Five mothers received P-CFT in a group setting within an Improving Access to Psychological Therapies (IAPT, now known as Talking Therapies for Anxiety and Depression) service in Manchester, UK. Following eight sessions, reductions in self-criticism and mood symptoms and improvements in levels of self-reassurance were noted for this very small clinical sample of only four mothers in the perinatal period. Although qualitative feedback from these participants indicated strong levels of acceptability, with this very small sample size and no control group, conclusions regarding the benefits of the intervention cannot be drawn.

## 4 Discussion

There is currently limited research to establish whether CFT or P-CFT improves maternal wellbeing. This Mini Review highlights that the evidence of Gilbert’s (13, 14) model in the perinatal population has so far only been applicable to mothers in non-clinical populations. The small-scale study of Griffiths and Virgin (30) is the only study that involved a clinical sample; albeit, the sample was very small in comparison to the other included studies (26–29). Furthermore, as the above interventions are primarily brief self-guided online interventions in comparison to Cree’s (15, 16) recommended 12-week-therapy, there is currently insufficient data to establish whether P-CFT would be beneficial to women receiving this particular intervention. As shown in Table 1, none of the identified studies in this Mini Review measured the mother-infant bond. Therefore, the effectiveness of CFT or P-CFT in improving the mother-infant relationship cannot be determined at this stage and requires further research, ideally using self-report measures and observer-rated measures.

In response to this, a mixed-methods study is currently being conducted across several NHS specialist perinatal mental health services that are offering P-CFT based on Cree’s (15, 16) adaptation, albeit within 10 to 12 sessions (36). This feasibility trial will explore whether P-CFT improves compassion-based and mood outcomes through the use of questionnaires, whether the intervention is acceptable through qualitative interviews, and whether P-CFT improves bonding through video-recorded and then independently coded parent-infant interactions. Whilst it will explore pertinent aspects of how best to implement P-CFT within perinatal mental health services (e.g., online delivery) and how best to collect meaningful outcome measures from women and on the mother-infant relationship, the planned study would also inform a future definite RCT.

## 5 Conclusion

These findings on Gilbert’s (13, 14) original CFT model from non-clinical perinatal populations have highlighted that P-CFT might be effective in improving PMH difficulties experienced by mothers. Although, the current evidence-base for P-CFT with clinical populations is very limited. Due to the potential adverse outcomes from PMH difficulties and a disrupted mother-infant relationship, it is important to examine whether P-CFT may optimize outcomes. It is apparent that further research is warranted, but it is anticipated that the planned mixed-methods study of P-CFT will provide some foundational understanding of its potential benefits.

## Author contributions

LM: Writing – original draft, Writing – review & editing. AW: Supervision, Writing – review & editing.

## Footnotes

1 Cree’s (15, 16) adaptation refers to the mother-infant relationship, however; this adaptation can also be applied to the father-infant relationship.
